# The Diverse Landscape of Negative Polarity Items: On the Use of German NPIs as Experimental Diagnostics

**DOI:** 10.1007/s10936-021-09793-0

**Published:** 2021-07-26

**Authors:** Katharina Schaebbicke, Heiko Seeliger, Sophie Repp

**Affiliations:** Institut für Deutsche Sprache und Literatur I, Universität zu Köln, Universitätsstraße 41, 50923 Köln, Germany

**Keywords:** Negative polarity items, NPI licensing, Graded acceptability, Nonveridicality, Rhetorical question

## Abstract

The goal of this study is to provide better empirical insight into the licensing conditions of a large set of NPIs in German so that they can be used as reliable diagnostics in future research on negation-related phenomena. Experiment 1 tests the acceptability of 60 NPIs under semantic operators that are expected to license superstrong, strong, weak, and nonveridicality-licensed NPIs, respectively: antimorphic (*not*), anti-additive (*no*), downward entailing (*hardly*), nonveridical (*maybe*, question). Controls were positive assertions. Cluster analysis revealed seven clusters of NPIs, some of which confirm the licensing categorization from the literature (superstrong and weak NPIs). Other clusters show unclear patterns (overall high or medium ratings) and require further scrutiny in future research. One cluster showed high acceptability ratings only with the antimorphic and the question operator. Experiment 2 tested whether the source of this unexpected distribution was a rhetorical interpretation of the questions. Results suggest that rhetoricity was not the sole source. Overall, the results show gradual rather than categorical differences in acceptability, with higher acceptability corresponding to stronger negativity. The paper provides the detailed results for the individual NPIs as a preliminary normed acceptability index.

## Introduction

Negative polarity items (NPIs) are frequently used in linguistic and psycholinguistic studies—either to study the phenomenon itself, or as a diagnostic for other linguistic phenomena, such as the scope of certain semantic operators, propositional vs. non-propositional negation, or question bias. NPIs are, generally speaking, words or phrases that are restricted to appearing in the scope of negation. For instance, the following sentence contains the NPI *much of* and would be unacceptable without the negation: Chris is *(not) much of a singer. Many NPIs are not only licensed by negation, but may also appear in certain non-negative environments, such as in questions or in the antecedent of conditionals.

The peculiar licensing conditions of NPIs have inspired a long tradition of research regarding their licensing behaviour. However, we are faced with two problems regarding the research on NPIs. Firstly, (theoretical) research often appears to limit its scope to the investigation of very few NPIs such as English *any*, *ever*, and *yet*, or German *jemals* ‘ever’, *sonderlich* ‘particularly’, or *keine müde Mark* ‘no red cent’. A closer look at the empirical picture, however, reveals that there is a vast landscape of NPIs in different shapes and forms. It is an open question whether all these NPIs can be accounted for by theories that rely on the analysis of a few token NPIs. For this reason, we believe that looking at a larger number of NPIs in a given language is an important step towards fully understanding the phenomenon of negative polarity sensitivity.

A second problem we face is that NPIs are sometimes used to explore or diagnose certain characteristics of negation or other potential licensing environments. However, usually experiments / linguistics tests in this area are not always backed up with control studies that explore the precise licensing conditions of the specific NPIs that are used as diagnostic. Yet, if we do not know the precise licensing conditions of an NPI, it cannot function as a reliable diagnostic, which leads to possible confounds. For example, NPIs as well as their counterparts, positive polarity items (PPIs; elements that cannot appear in the scope of negation) are often used to distinguish the type of negation present in polar questions with high negation such as *Doesn’t Mary like dogs?*. There is a line of research that claims that questions like the above are ambiguous between two readings: double-checking *p* (whether Mary likes dogs) and double-checking ¬*p* (whether Mary doesn’t like dogs) (Ladd, 1981). This ambiguity sometimes gets attributed to two different types of negation: propositional and non-propositional negation (Repp, 2013; Romero, 2015). NPIs and PPIs serve as diagnostics to distinguish the two readings: if an NPI can felicitously be inserted into the question, the negation must be propositional because NPIs require a type of propositional negation as a licensor. If a PPI can be inserted, the negation must be non-propositional because if the negation was propositional, it would anti-license the PPI. However, there is an obstacle in this approach: given that some NPIs can also be licensed in (non-negative) questions, it isn’t always clear whether a NPI is licensed by the negation or by the question itself in high-negation polar questions. So we cannot draw reliable conclusions with regard to the type of negation. In sum, we believe that broader knowledge on the exact licensing conditions of a larger set of NPIs can contribute to more stable empirical results in future research.

A look at the empirical landscape of German NPIs shows a hardly uniform picture with regard to various linguistic aspects. NPIs can be single words or groups of words, which do not seem to be very restricted with respect to what syntactic category they belong to, see (1)-(3). They also range from being semantically transparent (2), to being fully idiomatic (3).^1^

**Table:** 

(1)	Mia	freut	sich	nicht	**sonderlich**	*adverb (phrase)*
	Mia	is.happy	refl	not	particularly	
	‘Mia isn’t particularly happy.’					

**Table:** 

(2)	Sarah	weiß	die	Antwort	**beim**	**besten**	**Willen**	nicht	*prepositional phrase*
	Sarah	knows	the	answer	with.the	best	will	not	
	‘Even with the best will in the world Sarah doesn’t know the answer.’								

**Table:** 

(3)	Maria	**hat**	mit	Fußball	nichts	**am**	**Hut**	*verb phrase*
	Maria	has	with	football	nothing	on.the	hat	
	‘Maria doesn’t have any interest in football.’							

NPIs also show a lot of variation with respect to their licensing environments. Some NPIs, like *sonderlich* ‘particularly’, appear to be licensed by different negative elements, like *niemals* ‘never’ (4) and *kaum* ‘hardly’ (5), but not in questions (6). Other NPIs, like *zur Sache tun* ‘to matter’, however, can appear in questions without a problem (7).

**Table:** 

(4)	Paul	wird	niemals	**sonderlich**	gut	singen	können
	Paul	will	never	particularly	well	sing	can
	‘Paul will never be able to sing particularly well.’						

**Table:** 

(5)	Kaum	jemand	hier	kann	**sonderlich**	gut	singen
	hardly	anybody	here	can	particularly	well	sing
	‘Hardly anybody here can sing particularly well.’						

**Table:** 

(6)	^??^Kann	Paul	**sonderlich**	gut	singen?
	can	Paul	particularly	well	sing
	‘Can Paul sing particularly well?’				

**Table:** 

(7)	**Tut**	das	was	**zur**	**Sache**?
	does	this	something	to.the	cause
	‘Does this matter in any way?’				

This heterogeneous picture of NPIs, as illustrated here for German, shows that gaining a deeper understanding of the precise licensing conditions of NPIs is necessary, particularly if we want to use them as a diagnostic tool in other areas of linguistic research. Hence, the goal of the present study is just that: to systematically explore the licensing conditions of a great variety of NPIs in German.

We adopt the view by Zwarts (1986, 1998) and van der Wouden (1994) that NPIs largely fall into three groups of *superstrong*, *strong*, and *weak* negative polarity items, depending on whether they are licensed in antimorphic, anti-additive or downward entailing contexts. Additionally, we follow the view of Giannakidou (1997, 1998, 2002, 2011) and Zwarts (1995) that some nonveridical environments can also license NPIs. We present data from two acceptability rating experiments, where we explored the licensing conditions of 60 German NPIs. The goal of Experiment 1 was to classify these NPIs into superstrong, strong, weak and nonveridicality-licensed (*superweak*) NPIs by means of an acceptability study with the NPIs being rated in different licensing conditions. Experiment 2 explored some unexpected findings from Experiment 1: in Experiment 1, we found a cluster of NPIs that received high acceptability ratings only under the antimorphic operator *nicht* ‘not’ and in polar questions, which is not predicted by the above theories. Experiment 2 explored whether the unexpected rating pattern could be due to rhetorical interpretations of the questions. The present work builds on, and complements corpus studies which have provided a database of German NPIs through (semi)automated extraction of NPI candidates from corpora: the CoDII corpus (https://www.english-linguistics.de/codii/) (Sailer & Trawiński, 2006a, 2006b; Trawiński & Soehn, 2008; Trawiński et al., 2008).

The paper is organised as follows: in Sect. 2, we provide some theoretical background as well as background on previous quantitative research on NPIs. Experiments 1 and 2 are reported in Sects. 3 and 4, respectively. In Sect. 5, we discuss the findings from the two experiments in the light of previous theoretical accounts and conclude.

## Theoretical Background and Previous Quantitative Research

There is a variety of syntactic, semantic and pragmatic approaches to the question of NPI licensing. For the present study, we adopt the semantic views that have been put forward by van der Wouden (1994), Zwarts (1986, 1995, 1998) and Giannakidou (1997, 1998, 2002, 2011). Building on Ladusaw’s (1979) work on polarity sensitivity and downward entailment, Zwarts (1986, 1998) and van der Wouden (1994) suggest that NPIs largely fall into three classes, depending on what negative context they are licensed in. Van der Wouden (1994: 64) illustrates this for English NPIs with the following examples:

**Table:** 

(8)	a	Chomsky wasn’t **a bit** happy about these facts
	b	Chomsky didn’t talk about these facts **yet**
	c	Chomsky didn’t talk about **any** of these facts

**Table:** 

(9)	a	*No one was **a bit** happy about these facts
	b	No one has talked about these facts **yet**
	c	No one talked about **any** of these facts

**Table:** 

(10)	a	*At most three linguists were **a bit** happy about these facts
	b	*At most three linguists have talked about these facts **yet**
	c	At most three linguists have talked about **any** of these facts

The NPI *a bit* is only licensed by *not*, while the NPI *yet* is additionally licensed by *no one*, and the NPI *any* is licensed by the two aforementioned licensors as well as by *at most*. Zwarts (1986, 1998) and van der Wouden (1994) explain these different licensing patterns with the semantic properties of the contexts the NPIs can occur in. Zwarts (1986, 1998) suggests that there is a hierarchy of negative expressions, such that some negative contexts are stronger than others and consequently can license more NPIs. The strength of a negative context depends on the form of negation it contains, which impacts the logical properties of the respective sentence. We will not discuss these properties here, see van der Wouden (1994: 28 ff.) for details. The strongest type of negation is so-called *classical negation*, i.e. *not*. Contexts containing classical negation are *antimorphic*. The second strongest type of negation is so-called *minimal negation*, i.e. expressions like *nobody* and *never*. Contexts containing minimal negation are *anti-additive*. And thirdly, the weakest type of negation, *subminimal negation*, are *downward entailing* expressions such as *few* and *hardly*. NPIs like *a bit*, which are only licensed in antimorphic contexts, are called *superstrong NPIs*. NPIs like *yet*, which are licensed in antimorphic and anti-additive contexts, are called *strong NPIs*, and NPIs like *any*, which are additionally licensed in downward entailing contexts, are called *weak NPIs*.

While this threefold classification of NPIs has been successful in capturing the distribution of a large number of NPIs, it does not capture the fact that some NPIs can also appear in questions like (11):

**Table:** 

(11)	**Kann**	Paul	etwas	**dafür**?		
	can	Paul	something	for.that		
	‘Is that Paul’s fault?’					

There is no negation in (11), but still, the German NPI *dafürkönnen* ‘to be one’s fault’ is licensed. This cannot be easily explained in terms of downward entailment. Giannakidou (1997, 1998, 2002, 2011) observes that crosslinguistically NPIs often appear in a variety of environments that are not downward entailing, such as modal environments, imperatives, and questions. The common feature that these environments share is nonveridicality, which Giannakidou (1997, 1998, 2002, 2011) takes as the crucial notion that underlies NPI licensing. A propositional operator *F* is nonveridical if *F*(*p*) does not entail or presuppose that *p* is true in some individual’s epistemic model (Giannakidou, 1998). That is, in nonveridical sentences there is no truth commitment on the part of the speaker. The contrast in (12)(a–b) illustrates this: In the question in (12)(a), the speaker does not commit to the truth of the proposition *p* = *pick up* (*John*, *kids*), while in (12)(b) they do.

**Table:** 

(12)	a	Did John pick up the kids?
	b	John picked up the kids

We follow Giannakidou (1997, 1998, 2002, 2011) in assuming that not downward entailment, but nonveridicality is the final layer needed to explain the distribution of NPIs. Hence, we extend the superstrong/strong/weak NPI classification by Zwarts (1986, 1998) and van der Wouden (1994) accordingly, adding another layer to capture the NPIs that cannot only appear in downward entailing, but also in nonveridical contexts, see Table 1. For the purpose of the present study, we call that class *superweak NPIs* (cf. Hoeksema, 2012 for that term).

**Table 1:** Overview of NPI classes and their licensing contexts

Class of NPIs	Licensing contexts			
	Antimorphic	Anti-additive	Downward entailing	Nonveridical
Superstrong	+	−	−	−
Strong	+	+	−	−
Weak	+	+	+	−
Superweak	+	+	+	+

The goal of the present study will thus be to classify German NPIs according to the four licensing contexts summarized in Table 1. We expect that the NPIs can be grouped with respect to their licensing behaviour according to the different degrees of negativity. We expect to find that some NPIs need a stronger degree of negativity to be licensed than others. That is, there should be a group of NPIs that is only licensed in antimorphic contexts (superstrong NPIs), a group of NPIs that is additionally licensed in anti-additive contexts (strong NPIs), a group that is licensed in the two aforementioned contexts as well as in downward entailing contexts (weak NPIs), and a group that is licensed in all degrees of negative contexts including nonveridical ones (superweak NPIs). Furthermore, there should be no NPIs that are e.g. licit in a nonveridical context and an antimorphic context, but not in anti-additive and downward entailing contexts. Such a licensing pattern would be at odds with the idea of degrees of negativity corresponding to NPI strength.

As mentioned in the introduction, quantitative research particularly on German NPIs is so far relatively sparse. To our knowledge, experimental research on German NPIs has mostly focused on the processing of NPIs (Drenhaus et al., 2005; Saddy et al., 2004; Liu et al., 2019). Richter and Radó (2014) provide some insight into the licensing behaviour of German NPIs, but focus mainly on the strong/weak distinction, quantifier intervention and licensing by non-clausemate negation (i.e. cases where there is an NPI in an embedded clause which is licensed by a negation operator in the embedding clause). Seminal corpus-linguistic work in gaining a broad overview of the landscape of German NPIs has been done by Sailer and Trawiński (2006a, 2006b), Trawiński et al. (2008), and Trawiński and Soehn (2008). With the *Collection of Distributionally Idiosyncratic Items* (CoDII), they provide a multilingual database that contains data on lexical items with idiosyncratic distribution. The CoDII corpus provides amongst other things a list of 165 German NPI candidates that have been extracted from corpora, as well as syntactic and distributional information on those NPI candidates. The present study builds on, and complements information gathered in the CoDII corpus. The CoDII corpus classifies NPIs into superstrong, strong, and weak NPIs, depending on their distribution in the corpus. There are, however, some obstacles with the database due to methodological limitations of the corpus approach. Firstly, as is well known, the corpus approach can only find tokens that have been produced in a particular corpus, but not finding a particular NPI in a particular licensing context in a corpus does not mean it is unacceptable in this context. For example, there are no instances in the CoDII corpus of the German NPI *zu besagen haben* ‘to mean sth.’ licensed by negative verbs like English *to doubt*. However, intuitively, it seems to be acceptable in the scope of the negative verb *bezweifeln* ‘to doubt’:

**Table:** 

(13)	Ich	bezweifle,	dass	die	Zahlen	etwas	**zu**	**besagen**	**haben**
	I	doubt	that	the	figures	something	to	mean	have
	‘I doubt that these figures mean anything.’								

Secondly, the CoDII corpus so far does not distinguish between different forms of questions, most notably between positive and negative polar questions. As a consequence, if an NPI appears in a negative polar question in the corpus, it is not clear whether that particular NPI is licensed by the question or by the negation, leading to possible confounds in the NPI classification. Another issue is the licensing of NPIs in rhetorical questions, which has been argued to be subject to more specific conditions (van Rooy, 2003, see Experiment 2 for discussion). Rhetorical questions are not distinguished from non-rhetorical questions in the CoDII corpus either. For these reasons, we believe that a multi-methodological approach to German NPIs employing experimental methods to complement the quantitative data obtained in the CoDII corpus will substantially broaden our understanding of NPI licensing. Systematic quantitative testing of the NPI candidates found in CoDII in controlled acceptability judgement experiments is a first step in this direction.

## Experiment 1

The goal of Experiment 1 is to obtain a reliable classification of a set of German NPIs from the CoDII database into superstrong, strong, weak, and superweak NPIs, following the NPI licensing theories by Zwarts (1986, 1998), van der Wouden (1994), and Giannakidou (1997, 1998, 2002, 2011). To achieve this, we conducted an acceptability judgement study of a set of 60 German NPIs, which were embedded in sentences in six different licensing conditions that should allow the classification of these NPIs into the aforementioned classes. The NPIs were selected from the CoDII corpus on the basis of the following selection criteria: highly idiomatic NPIs were excluded because they show little to no syntactic flexibility so that alternations in licensing are virtually non-existent. For example, the NPI *auf keine Kuhhaut gehen* (lit. on no cow’s skin go, ‘to beggar belief’) is an idiom NPI that appears to be frozen with its licensor *keine* ‘no’ and doesn’t allow for any other licensor. Furthermore, building experimental items with idioms of this sort is not easy beyond trivial variations e.g. by changing the subject DP with them. Additionally, we excluded semantic duplicates such as *zu deuteln sein* und *sich deuteln lassen* (lit.: to interpret be vs. refl interpret let, ‘to leave room for interpretation’), choosing one of the variants only. Obviously antiquated phrases were left out as well because we presumed that they would receive a low acceptability rating due to not being frequent anymore. Furthermore, we excluded verbal NPIs that require embedded sentences as a complement in order to keep the design of the experimental items as uniform as possible. And lastly, we excluded the NPI *jemals* ‘ever’ from the study because in contexts with sentential negation it has to fuse with the negation operator (neg + *ever*) and becomes a licensor itself: *niemals* ‘never’.

The 60 remaining NPIs had different morphosyntactic and lexical characteristics. Since these characteristics in principle might have an influence on the licensing conditions, we grouped the NPIs into ad-hoc groups based on their morphosyntactic characteristics: [1] PPs and AdvPs occurring as adverbials (e.g. *im Geringsten*, lit.: in.the slightest, ‘in the slightest’) (*n* = 8), [2] NPs occurring as subjects or objects (e.g. *Pieps* ‘beep’) (*n* = 9), [3] PPs or infinitives serving as predicates in copula sentences with the verb *sein* ‘to be’ (e.g. *bei Trost sein*, lit.: with consolation be, ‘to be in one’s right mind’) (*n* = 5), [4] combinations of a modal verb and a lexical verb in the bare infinitive (e.g. *ausstehen können*, lit.: stand can, ‘to be able to stand sth./sb.’) (*n* = 14), [5] a modal verb taking a *to*-infinitive (*brauchen* ‘need’), or combining with an adverb (*anders können*, lit. different can, 'to have a choice') (*n* = 2), [6] combinations of the verb *lassen* ‘to let’ with a (reflexive) lexical verb in the bare infinitive (e.g. *sich reinreden lassen*, lit.: refl interfere let, ‘to be swayed’) (*n* = 8), and [7] other lexical verbs or VPs with flexible arguments (e.g. *sich scheren um* lit.: refl care about, ‘to care about sth./sb.’) (*n* = 12). Two NPIs did not fit in any of these groups. Additionally, there were two groups of NPIs that stood out from the perspective of the negation marker they require: the first group were nominal NPIs that cannot appear in the scope of sentential negation *nicht* ‘not’, but must instead appear with the negative indefinite determiner *kein* ‘no’ (= *kein* group, *n* = 10), for example *keine Menschenseele* ‘no living soul’, because the negative operator and the indefinite determiner fuse: neg + *ein* ‘a’ = *kein*.^2^ The other group were verbal NPIs that also cannot appear in the scope of sentential negation, but instead require the negative pronoun *nichts* ‘nothing’ as one of their arguments (= *nichts* group, *n* = 8), for example *dafürkönnen* ‘to be one’s fault’.

### Method

#### Materials, Design and Procedure

The 60 German NPIs were tested in six conditions, see Table 2 for illustration. To keep the design as uniform as possible, nonverbal NPIs were inserted in sentences with transitive predicates. For verbal NPIs, the argument structure of the predicate of course depended on the NPIs themselves. Condition [1] was the antimorphic condition, with the licensor *nicht* ‘not’. The word *nicht* in German is an adverb (but sometimes is considered a particle), which may appear in various sentence-medial positions. In approximately a third of the target sentences (22/60), it appeared between a definite object and the clause-final transitive verb. For verbal NPIs, the position of *nicht* usually was directly before the clause-final verb. As mentioned above, the NPIs in the *kein* group and the *nichts* group cannot appear in the scope of *nicht* ‘not’.^3^ Thus, the NPIs in these groups cannot be classified as superstrong NPIs due to their morphosyntactic restrictions. To nevertheless obtain an insight into their properties regarding the precise anti-additive licensing patterns, these NPIs were tested both in the anti-additive and in the ‘antimorphic’ conditions with their required anti-additive licensor but this licensor appeared in different syntactic positions. In the ‘antimorphic’ condition, the NPIs in the *kein* group were direct objects and were directly preceded by their licensor *kein*, so that the syntactic pattern was subj – v – *kein* obj.npi. The NPIs in the *nichts* group are idiomatic to a certain extent, in that the position of the negative licensor *nichts* is fixed and can either be an object or a subject, depending on the NPI. In the ‘antimorphic’ condition these NPIs appeared with the licensor *nichts* in the required position.

**Table 2:** Sample items and predictions per condition of Experiment 1

Condition		Licensor	Sample target sentence	Prediction: acceptable NPIs
[1]	Antimorphic	*nicht* ‘not’	*Peter mag die Aufgabe nicht sonderlich* Peter likes the task not particularly ‘Peter doesn’t particularly like the task.’	All NPIs
[2]	Anti-additive	*kein* ‘no’	*Kein Mitarbeiter mag die Aufgabe sonderlich* no employee likes the task particularly ‘No employee particularly likes the task.’	Strong, weak, superweak NPIs
[3]	Downward entailing	*kaum* ‘hardly’	*Kaum ein Mitarbeiter mag die Aufgabe sonderlich* hardly an employee likes the task particularly ‘Hardly any employee particularly likes the task.’	Weak, superweak NPIs
[4]	Nonveridical modal operator	*vielleicht* ‘maybe’	*Vielleicht mag Peter die Aufgabe sonderlich* maybe likes Peter the task particularly ‘Maybe Peter particularly likes the task.’	Superweak NPIs
[5]	Nonveridical question operator	Q	*Mag Peter die Aufgabe sonderlich?* likes Peter the task particularly ‘Does Peter like the task particularly?’	Superweak NPIs
[6]	Positive assertion	none	*Peter mag die Aufgabe sonderlich* Peter likes the task particularly ‘Peter likes the task particularly.’	None

Condition [2] was the anti-additive condition with the licensor *kein* ‘no’, which is a quantificational determiner. In the target sentences, it was part of a negative quantifier in the subject position. For the NPIs of the *kein* group, *kein* also appeared in this position and did not directly precede the NPI as in the ‘antimorphic’ condition. The anti-additive condition for the *nichts* group was also analogous to the other NPIs in this condition, with *kein* as part of the subject.

Condition [3] was downward entailing with the licensor *kaum* ‘hardly’. Like *kein*, *kaum* is a quantificational determiner, and it appeared as part of a quantifier in the subject position of the target sentences. There were two nonveridical conditions.

Condition [4] was nonveridical with the nonveridical modal operator *vielleicht* ‘maybe’, which is an adverb in German. This condition was added in order to explore whether nonveridicality in general can license certain German NPIs, or whether NPI licensing in German is limited to particular nonveridical operators. Note that there is a homonymous *vielleicht* that is a modal particle and has a different meaning from the adverb. In declarative sentences, this meaning is an emphatic meaning: *Peter war vielleicht nervös*, lit. Peter was part nervous, ‘Peter was really nervous’. We ensured that *vielleicht* in the experimental sentences was an adverb with the nonveridical meaning ‘maybe’ by placing it in the sentence-initial position in verb-second declaratives, where modal particles cannot occur on their own.

Condition [5] was the other nonveridical condition, where the NPI occurred in a positive polar question, i.e. the nonveridical operator was the question operator.

Condition [6] served as a control condition. Here, the NPI occurred in a positive assertion, i.e. there was no licensing operator.

Every target sentence was preceded by a short context story. This was done in order to prevent degraded ratings due to out-of-the-blue presentations of the stimuli and to help participants get the intended reading of the respective NPIs even in conditions without a licensor. All items were constructed so that the target sentence was uttered by a protagonist from the context story. Across conditions, the context stories were kept as stable as possible, see Table 3 for an example. However, as the main goal in creating the context stories was to provide a context that facilitates the interpretation of the NPIs in every condition, i.e. even in conditions where they might not be licensed, and to make the target sentences as contextually plausible as possible, there was some variation in the length of the stories, which did not correlate with condition.

**Table 3:** Context stories for the six conditions of the sample items in Table 2 in Experiment 1

Context story	Target
*Peter soll für seine Firma die Weihnachtsfeier organisieren. Er hat das letztes Jahr schon gemacht und hat deswegen jetzt schlechte Laune. Seine Kollegin sagt zur Chefin:* Peter was told to organise the Christmas party for his company. He did that last year already, and is not happy about the task. His colleague tells the boss:	[1]
*Peters Chef sucht jemanden, der für die Firma die diesjährige Weihnachtsfeier organisiert. Er überlegt, welcher seiner Mitarbeiter vielleicht Lust auf die Aufgabe hätte, und fragt Peter. Peter weiß, dass die Aufgabe nicht sehr beliebt ist, und antwortet:* Peter’s boss is looking for someone to organise the company Christmas party. He does not know which employee is up for the task, and asks Peter about it. Peter knows that the task is not very popular, and replies:	[2]
*Peters Chef sucht jemanden, der für die Firma die diesjährige Weihnachtsfeier organisiert. Er überlegt, welcher seiner Mitarbeiter vielleicht Lust darauf hätte, und fragt Peter. Peter weiß, dass die Aufgabe nicht sehr beliebt ist, und antwortet:* Peter’s boss is looking for someone to organise the company Christmas party. He does not know which employee is up for the task, and asks Peter about it. Peter knows that the task is not very popular, and replies:	[3]
*Peter soll für seine Firma die Weihnachtsfeier organisieren. Er hat das die letzten Jahre schon gemacht. Seine Kollegen sind deswegen überrascht, dass er das schon wieder machen soll. Eine Kollegin spekuliert:* Peter was told to organise the Christmas party for his company. He had had to do that task for a few years already. Hence, his colleagues are surprised that the task fell on him again. One of his colleagues speculates:	[4]
*Peter soll für seine Firma die Weihnachtsfeier organisieren. Er hat das letztes Jahr schon gemacht. Deswegen ist seine Kollegin überrascht und fragt die Chefin:* Peter was told to organise the Christmas party for his company. He did that last year already. Hence, a colleague of his is surprised and asks the boss:	[5]
*Peter soll für seine Firma die Weihnachtsfeier organisieren. Er hat das letztes Jahr schon gemacht und hat deswegen jetzt gute Laune. Seine Kollegin sagt zur Chefin:* Peter was told to organise the Christmas party for his company He did that last year already, and is happy to do it again. His colleague says to the boss:	[6]

The number of lexicalisations per NPI was restricted to one because of the high number of NPIs, that is each NPI was tested with one experimental sentence per condition: a high number of lexicalisations would have been unmanageable in a study aiming at obtaining a broad overview of the landscape of German NPIs.

The predictions for the six experimental conditions were as follows: **superstrong NPIs** should be rated as acceptable only in the antimorphic condition [1]. **Strong NPIs** should additionally receive high acceptability ratings in the anti-additive condition [2]. **Weak NPIs** should be acceptable in the two aforementioned conditions as well as in the downward entailing condition [3]. **Superweak NPIs** should be acceptable in the antimorphic [1], anti-additive [2], downward entailing [3] and the question condition [5]. If nonveridicality in general can license superweak NPIs in German, then the NPIs that are rated as acceptable in the question condition should also receive a high acceptability rating in the nonveridical condition [4] with the modal operator *vielleicht* ‘maybe’. In the positive-assertion condition [6], all NPIs should receive a low acceptability rating. If an item received a high rating in the positive condition, that would suggest that this item is in fact not an NPI or is polysemous with a non-NPI.

In addition to the experimental items, there were 20 filler items which consisted of five positive assertions, five negative assertions, five positive questions and five negative questions. Half of the fillers were expected to be rated as acceptable, and half of the fillers were expected to be rated as unacceptable. The filler items were constructed analogously to the target items, such that they were embedded in a context story, with one of the protagonists from the context story uttering the filler sentence.

With 60 NPIs in 6 conditions, there were 360 experimental items overall in Experiment 1. These experimental items were evenly distributed across 6 experimental lists. The 6 lists were subdivided into 3 questionnaires each so as to avoid a decline in attention and exhaustion of the participants. In sum, there were thus 18 different questionnaires, each of which contained 20 experimental items plus the 20 filler items. Experimental and filler items were pseudo-randomised in each questionnaire. The task was a pen-and-paper task, allowing participants to go back and forth between items. Participants were asked to read the context story and the target sentence and then to rate the naturalness of the target sentence on a 7-point scale, indicating how natural the sentence sounded in the given context. The endpoints of the scale were labelled *sehr natürlich* ‘very natural’ and *sehr unnatürlich* ‘very unnatural’. In the analysis, these endpoints were translated into the numbers 7 and 1, respectively, with the scale points in between corresponding to the numbers 2–6.

#### Participants

400 monolingual native speakers of German took part in Experiment 1 (164 m, 235 f, 1 d; age 16–85 (*m* = 29.65, *SD* = 13.21)). They were mainly from Cologne, Germany, and surrounding areas. The data of 40 participants were discarded because they were bilingual with a daily use of both languages or because they did not have German as a native language, leaving the data from 360 participants for analysis, so that there were 20 data points per NPI per condition. 200 participants were recruited from the general population by 20 students taking part in a university class on negation as part of their course assignment. 200 participants were recruited on campus by three research assistants at the University of Cologne, and received 3 € payment for their participation. The questionnaires were filled in in informal settings (home, quiet room on campus). Filling out the questionnaires took 15 to 20 min on average.

### Results and Discussion

Of the 7200 data points we obtained, 2.7% were discarded because participants did not give the predicted judgements for the filler items, or because they forgot to judge an item. As for the filler criterion, the data of participants who judged more than 30% of the acceptable fillers as unnatural (≤ 3) and unacceptable fillers as natural (≥ 5) were removed from the dataset.

**Fig. 1:**
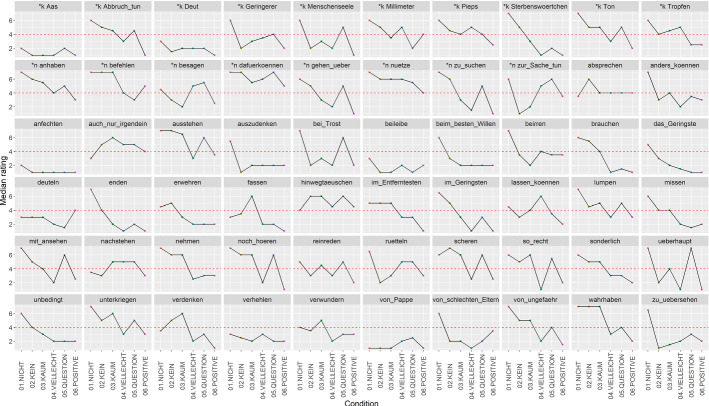
Median ratings of NPIs per condition. Row 1 contains the 10 NPIs of the ‘kein’ group, as marked by *k. Row 2 contains the 8 NPIs of the ‘nichts’ group, marked by *n. The subsequent NPIs are sorted alphabetically

To analyse the data, we performed descriptive statistics including visual inspection and a cluster analysis (R package cluster, Maechler et al., 2019). Cluster analysis is well-suited for exploratory data analysis: it allowed us to identify NPIs patterning together in their licensing behaviour. Figure 1 shows the median rating of all 60 NPIs per condition. Note that due to space reasons, some NPIs in Fig. 1 are abbreviated. See Appendix for the full list of (unabbreviated) NPIs; they can also be accessed in the CoDII database via https://www.english-linguistics.de/codii/codiinpi/de/list-complete.xhtml.^4^

As Fig. 1 shows, there is a lot of variation in the licensing patterns of the NPIs in Experiment 1. At first glance, some NPIs, like *auszudenken sein* (lit.: make.up be, ‘to be conceivable’ (*auszudenken* in Fig. 1)) and *beim besten Willen* (lit.: at.the best will, ‘with the best will in the world’) seem to act like superstrong NPIs, with high ratings in the antimorphic *nicht* condition and low ratings in all other conditions. For other NPIs, e.g. *zur Sache tun* (lit.: to.the thing do, ‘to matter’), the pattern is far from clear.

**Fig. 2:**
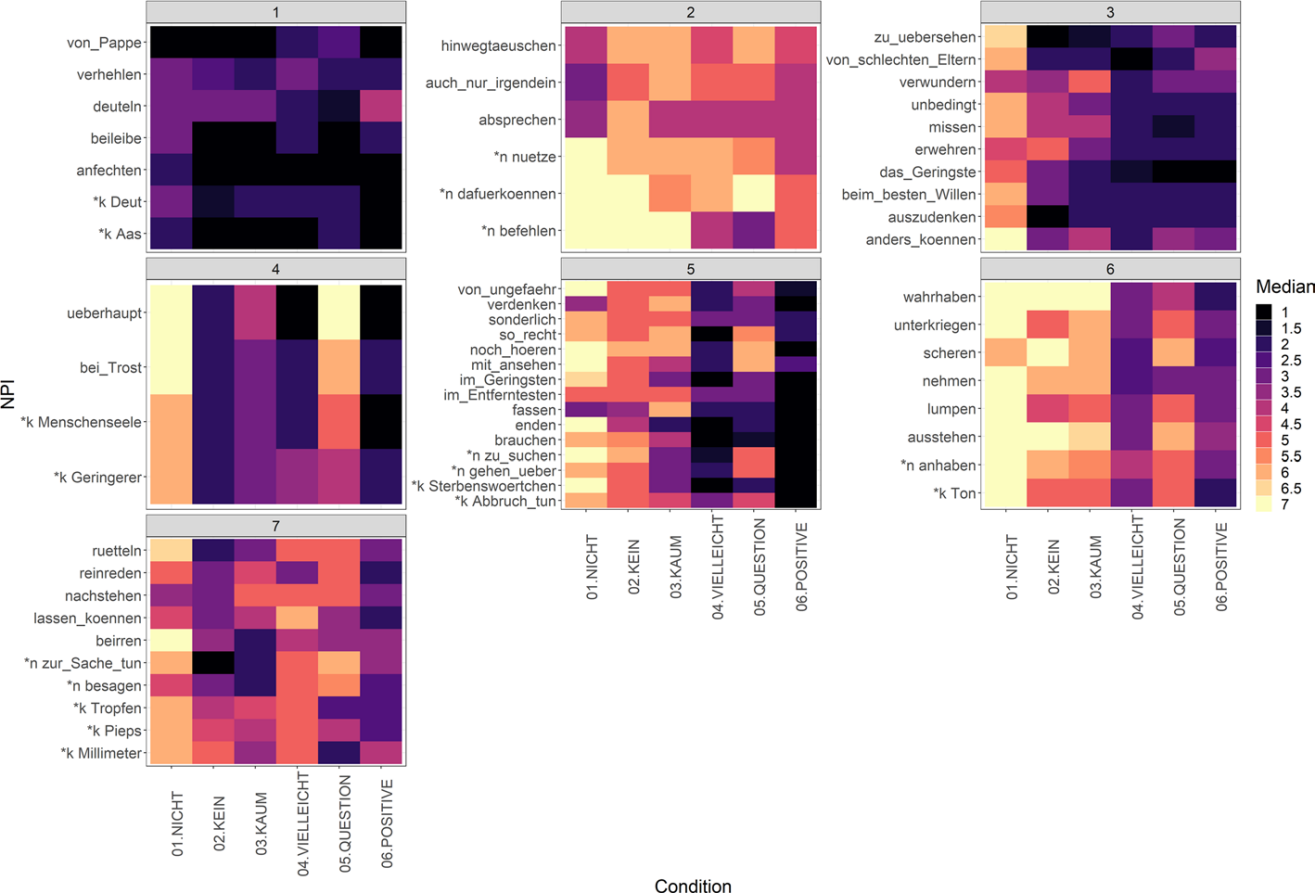
Median rating per NPI per condition in seven clusters, with lighter colour corresponding to higher medians (Color figure online)

On the basis of the cluster analysis, we identified the seven clusters shown in Fig. 2. None of these clusters or other potential smaller clusters showed an influence of the morphosyntactic and/or lexical characteristics of the NPIs. The only clear pattern that emerged upon visual inspection was the following: in the *kein* group, there is a notable decline in acceptability between the antimorphic condition, in which the NPIs from the *kein* group were directly preceded by their licensors, and the anti-additive condition, in which the licensor *kein* was part of the subject, but the NPIs were in object position (subj – v – *kein* obj.npi vs. *kein* subj – v – obj.npi). This shows that the nominal NPIs in the *kein* group are idiomatic to the extent that they have to be preceded by their licensor directly. When the same licensor appears in a position higher up in the sentence, the acceptability is notably degraded. Apart from this, neither the *nichts* group nor the *kein* group nor the morphosyntactic groups described in Sect. 3 showed uniform licensing behaviour and formed a cluster.

In cluster [1], all NPIs received low ratings in all conditions. A probable explanation for this is low frequency: the NPIs in cluster [1] appear to be antiquated and/or not widely used. For instance, an analysis of the development of the word frequency of the phrase *von Pappe sein* (lit.: of cardboard be ‘to be of sound quality’), as inspected with the *DWDS Wortverlaufskurven* ‘word timelines’ (https://www.dwds.de/d/ressources#wortverlauf), indicates that the frequency of that phrase has declined considerably in the last 31 years (independently of its concrete meaning). The word *Deut* ‘brass farthing’ has been infrequent (1 per million tokens) for the last 250 years. Furthermore, since most participants were from the area of Cologne, dialectal variation could also play a role, which needs to be investigated in future research.

Cluster [2] shows relatively high ratings overall, with only a slight decline in ratings with declining negativity. In fact, in the positive assertion condition 6 the NPIs of cluster [2] received median ratings of 4 and higher, i.e. they were perceived to be acceptable rather than unacceptable. This finding suggests that the items in cluster [2] are not actually NPIs. Still, for most items in this cluster, the positive condition is the condition with the lowest rating. This effect might be due to these items often occurring with negation but not being actually NPIs, that is, we might be observing a frequency effect. For instance, a corpus search in the DWDS corpus (https://www.dwds.de/) revealed that examples without licensor can readily be found for *befehlen lassen* (lit. oneself sth. order let, ‘to be bossed around’) or for *hinwegtäuschen können* (lit. deceive can, ‘to be able to obscure the fact that …’), *absprechen können* (lit. deny can, ‘not to be able to deprive sb. of sth.’) (both more often without *können*). This issue needs to be investigated in future research.

Cluster [3] shows high acceptability ratings in condition 1, and a clear drop in acceptability in all other conditions, suggesting that the NPIs in cluster [3] are only licensed by the antimorphic licensor *nicht* ‘not’, and are thus superstrong NPIs, as described by van der Wouden (1994) and Zwarts (1986, 1998).

Cluster [4] shows high acceptability ratings in the antimorphic condition and in the question condition, and low ratings in all other conditions. This pattern is highly unexpected from the perspective of the superstrong/strong/weak/superweak distinction: if NPIs receive high ratings in questions, they should also receive high ratings in anti-additive and downward entailing contexts. Closer inspection of the individual NPIs in cluster [4] suggests two possible explanations for the unexpected finding. Firstly, the NPI *überhaupt* ‘at all’ appears to have an obligatory reading as a modal particle in questions rather than the adverb NPI reading we intended. The NPI *überhaupt* intensifies the negation: *Das hat sie überhaupt nicht gesagt* (lit. that has she at.all not said ‘She didn’t say that at all.’). The modal particle *überhaupt* has a different meaning in questions. It signals that the speaker wants to question a proposition (the prejacent of the question *überhaupt* is inserted in) in the common ground: *Hat sie das überhaupt gesagt?* (lit. has she that part said ‘Did she even say that?’), or it signals the speaker’s displeasure with something the question refers to: *Was will der überhaupt?* (lit. what wants the part, ‘What does he even want?’). Arguably, both these meanings differ significantly from the negation-intensifying NPI *überhaupt*. It seems that the modal particle reading is available in the question we tested in Experiment 1, so we assume that the high ratings are due to this reading (whether or not the two readings should be analysed separately or not is still debated, see Anderssen (2006) and Rojas-Esponda (2014) for discussion). Secondly, it is possible that the other question items in cluster [4] were interpreted as rhetorical questions. For instance, the following item has a rhetorical flavour: *Ist Philipp bei Trost?* (lit. is Philipp with consolation). On the rhetorical reading, it translates to ‘Is Philipp insane?’. This reading may have overridden the intended neutral reading ‘Is Philipp sane?’. Despite our attempts to make the questions as neutral as possible with the context stories, it is possible that participants interpreted the questions in cluster [4] and possibly other questions with high ratings in Clusters [5] and [6] (see below) as rhetorical. Since rhetoricity might have an influence on NPI licensing (van Rooy, 2003), we explored this potential confound in Experiment 2, see Sect. 4.

Cluster [5] and cluster [6] both show low ratings in the positive condition as well as in the modal operator nonveridical condition. This suggests that the modal nonveridical operator *vielleicht* cannot license NPIs in German. Apart from this clear pattern, cluster [5] shows a lot of variation. Nevertheless, visual inspection shows that there is a decline in acceptability ratings between the antimorphic, anti-additive, and downward entailing condition in 10 out of 15 NPIs in this cluster. There is often no clear cut between acceptability ratings, i.e. the differences between the different licensors are gradual. For instance, the NPI *mit ansehen können* (*mit ansehen* in the figure, lit. with behold can ‘to be able to bear sth.’) received median acceptability ratings of 7, 5, and 4 in the antimorphic, anti-additive, and downward entailing condition respectively. It is difficult to decide on the threshold for the classification of NPIs given such minimal median differences. We will come back to this issue further below. As for the seemingly idiosyncratic behaviour of other NPIs in cluster [5], further experimental testing with different experimental sentences is required. The NPIs in cluster [6] received overall high ratings in the antimorphic, anti-additive, and downward entailing condition, suggesting that these NPIs are weak NPIs. For the reasons described above, i.e. the high acceptability ratings in questions possibly being due to rhetorical interpretations, the question condition was examined again in Experiment 2. If the high question ratings are not due to rhetorical interpretations, the NPIs with high ratings in questions in cluster [6] can be classified as superweak NPIs.

In Cluster [7], acceptability ratings are highest in the antimorphic condition, but all other conditions show medium to low ratings. Since the positive condition received the lowest ratings overall, the items in cluster [7] do in fact appear to be NPIs. Interestingly, the anti-additive (*kein*) condition received lower ratings than the downward entailing (*kaum*) condition in 6 out of 10 NPIs in cluster [7]. This shows that the tendency of lower ratings with declining negativity is not present in all NPIs in Experiment 1. It is possible that this finding can be attributed to the design of the items. Recall that the target sentences in the downward entailing condition contained the licensor *kaum* ‘hardly’ as a quantificational determiner in the subject. It seems that some NPIs received a low acceptability rating in the downward entailing condition, but are acceptable with *kaum* as an adverbial quantifier. For example, the NPI *etwas zur Sache tun* (lit. sth. to.the thing do ‘to matter’) received a median rating of 2 with *kaum* as a quantificational determiner (see the experimental item in (14)), but appears to be acceptable with *kaum* as an adverbial quantifier, as shown in (15):

**Table:** 

(14)	Kaum	ein	Ort	**tut**	**etwas**	**zur**	**Sache**
	hardly	a	place	does	something	to.the	thing
	‘Hardly any place matters.’						

**Table:** 

(15)	Letzteres	**tut**	kaum	**etwas**	**zur**	**Sache**
	the.latter	does	hardly	anything	to.the	thing
	‘The latter hardly matters.’ From *Süddeutsche Zeitung*, 15.9.2020, Article *Mit Kippa und Lederhosen* ‘With kippa and lederhosen’ (https://www.sueddeutsche.de/muenchen/ausstellung-mit-kippa-und-lederhosn-1.5032394)					

From the perspective of van der Wouden (1994) and Zwarts (1986, 1998) it is rather unexpected to find an acceptability difference between the quantifying determiner and the adverbial quantifier *kaum* with the same NPI: both are downward entailing, as can be shown by checking the entailment relations: (16)(a) entails (16)(b), and (17)(a) entails (17)(b).

**Table:** 

(16)	a	Kaum	ein	Kind	isst	gern	Gemüse	b	Kaum	ein	Kind	isst	gerne	Spinat
		hardly	a	child	eats	gladly	vegetables		hardly	a	child	eats	gladly	spinach
		‘Hardly any child likes to eat vegetables.’							‘Hardly any child likes to eat spinach.’					

**Table:** 

(17)	a	Meine	Kinder	essen	kaum	Gemüse	b	Meine	Kinder	essen	kaum	Spinat
		my	children	eat	hardly	vegetables		my	children	eat	hardly	spinach
		‘My children hardly eat vegetables.‘						‘My children hardly eat spinach.’				

One difference between the two quantifiers is that the quantifying determiner quantifies over individuals, and the adverbial quantifier quantifies over situations. However, since both are downward entailing, differences in NPI licensing are not expected from the perspective of van der Wouden (1994) and Zwarts (1986, 1998). Why exactly the two different quantifier types seem to differ in NPI licensing needs to be established in future research.^5^

To summarize, in Experiment 1 we found some NPIs that match the predictions made with regard to the literature: superstrong NPIs were expected to only be licensed in the antimorphic condition [1), which is what we found in cluster [3]. Weak NPIs were expected to be licensed in the antimorphic, anti-additive, and downward entailing condition [1–3], and superweak NPIs were additionally expected to be licensed in the question condition [5]. The NPIs found in cluster [6] match these predictions. Additionally, Experiment 1 has established that the nonveridical modal operator *vielleicht* ‘maybe’ does not license superweak NPIs in German. Generally, ratings in the *vielleicht* condition were as low as ratings in the positive assertion condition. Thus, we tentatively conclude that it is not nonveridicality that licenses superweak NPIs in German, but that questions have specific licensing properties. This issue needs closer scrutiny in future research as there are other nonveridical environments, such as conditionals, in which NPIs have been observed to occur. Still, our results do not exclude the possibility that questions have specific NPI licensing properties that are not related to nonveridicality.

Overall, the acceptability ratings are not as categorical as could have been expected from a theoretical perspective, with clear cuts between high vs. low acceptability, but rather graded. Although this is not an unusual finding in experimental testing, the differences between the different licensors often are very small. The size of these differences needs to be quantified in future experiments that are designed to allow inferential testing. Nevertheless, even if the differences are small, the graded acceptability seems to reflect Zwarts’s (1986, 1998) hierarchy of negation: the more negative the context, the higher the ratings. A very peculiar licensing pattern in this regard was that of cluster [4], with high acceptability ratings only in the antimorphic and the question condition. As mentioned above, this licensing pattern is not in accordance with the idea of gradual negativity corresponding to gradual NPI strength. Since item analysis suggested that the high acceptability ratings in questions in cluster [4] could stem from rhetorical readings, Experiment 2 was conducted to determine whether rhetoricity contributed to high acceptability ratings in questions in Experiment 1.

## Experiment 2

The primary goal of Experiment 2 was to find out whether participants interpreted the questions that received a high acceptability rating in Experiment 1 as rhetorical, that is, as a question that a speaker does not ask because they want to know the answer, but because they want to express that the answer to the question is obvious. Rhetorical questions have been suggested to be, or to be similar to, assertions (e.g., Sadock, 1971, 1974; Han, 2002; but see Caponigro & Sprouse, 2007). They express that the prejacent of the question—or, more typically, its negation—is true.

According to van Rooy (2003), there is a certain type of NPI that only appears in rhetorical questions: NPIs that denote minimal values such as *a drop* in *He doesn’t drink a drop* (so-called *minimizer NPIs*; or ‘*even* NPIs’ in van Rooy’s (2003) analysis). Non-minimizer NPIs, in contrast, can appear in neutral questions. Van Rooy (2003) suggests that sentences containing a minimizer NPI have a similar presupposition to sentences with *even*: that there are salient alternatives to their denotation, which are relevant for their interpretation. Questions containing minimizer NPIs presuppose that the question is already settled for the salient alternatives, but not for the minimal value. For example, the question *Does Paul drink a drop?* presupposes that the question whether Paul drinks anything more than the minimal value is already settled, i.e. that Paul does not drink alcohol regularly. Hence, the question can only be asked to settle whether Paul does not drink any alcohol at all, or whether there is an exception to it, namely the minimal amount. According to van Rooy (2003), this is the rhetorical effect that minimizer NPIs have in questions: to settle whether nothing at all applies, or whether the minimal amount applies, and since the minimal amount is practically nothing, the question is interpreted as rhetorical.

From the above we can derive the following predictions for Experiment 2: if rhetoricity was the source of high acceptability ratings in Experiment 1, we expect all the questions with high acceptability ratings to receive high rhetoricity ratings in Experiment 2 as well. That is, we should find a positive correlation between acceptability and rhetoricity. In addition, we can make a second prediction: since van Rooy (2003) suggests that minimizer NPIs appear only in rhetorical questions, the questions with minimizer NPIs from Experiment 1 should receive high rhetoricity ratings in Experiment 2. Non-minimizer NPIs, however, should receive low rhetoricity ratings, because according to the theory they are not associated with rhetoricity. To establish whether these hypotheses hold, the 60 question items from Experiment 1 were tested for acceptability and rhetoricity in Experiment 2.

### Method

#### Materials, Design and Procedure

The 60 experimental items were distributed across two lists. In addition to the experimental items, there were 30 filler items, consisting of positive and negative questions embedded in context stories. Each participant saw 30 experimental items and 30 filler items. Like in Experiment 1, participants were asked to read the context story and the target sentence and then judge the acceptability of the item on a 7-point scale, with the endpoints labelled *sehr natürlich* ‘very natural’ and *sehr unnatürlich* ‘very unnatural’. In addition, the level of rhetoricity was measured: participants were asked to judge on another 7-point scale how likely they thought it was that the person asking the question in the story already knew its answer. The endpoints of this scale were labelled *sehr unwahrscheinlich* ‘very unlikely’ (1) and *sehr wahrscheinlich* ‘very likely’ (7). Both scales were presented on the same page as the experimental items, with the acceptability scale above the rhetoricity scale. To ensure that participants read the contexts and target sentences carefully, participants had to answer a control question about the context or the contents of the target sentence after each item, after they had made the judgements. Experiment 2 was conducted as an online questionnaire using SoSci Survey (Leiner, 2019) and made available via www.soscisurvey.de.

#### Participants

In Experiment 2, 82 monolingual native speakers of German were tested (64 f, 18 m), aged 19–43 (*m* = 22.54, *SD* = 4.05) mainly from Cologne, Germany, and surrounding areas. The participants were recruited from an introductory linguistics class at the University of Cologne and received course credit for taking part in the experiment. They filled out the questionnaires online from their homes. They could not go back and forth between items. On average, filling out the questionnaire took 40 min.

### Results and Discussion

Again, participants who judged more than 30% of the acceptable fillers as unnatural and unacceptable fillers as natural were removed from the dataset. Additionally, participants who gave the wrong answer to more than 1/6 of the control questions were removed. Overall, 5 participants were excluded from the dataset, resulting in 6.09% data loss.

Experiment 2 largely replicated the results for acceptability obtained in Experiment 1. Out of 60 items in Experiment 1 and 2 respectively, 19 had the same median (difference = 0); 8 had a difference in medians of 0.5; 19 had a difference in medians of 1; 3 had a difference in medians of 1.5; 9 had a difference in medians of 2; 2 had a difference in medians of 2.5. That is, for 46 out of 60 NPIs the difference between medians in Experiment 1 and 2 was ≤ 1. For the descriptive statistics we focused on the questions that received a high acceptability rating (median ≥ 5). The reasoning for this was that it seems unlikely that participants can have clear intuitions about the rhetoricity of a question if they deem that question unacceptable.

**Fig. 3:**
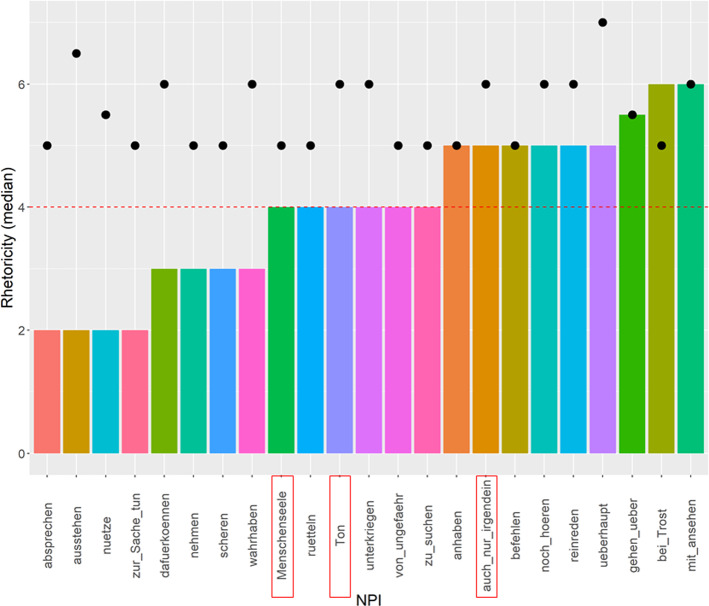
Questions with median acceptability ≥ 5 ordered by median rhetoricity, as indicated by the bars. The black dots indicate the median acceptability rating. Red boxes mark minimizer NPIs (Color figure online)

Figure 3 shows the rhetoricity results for questions that received a median acceptability rating of ≥ 5. It suggests that rhetoricity did not contribute much to acceptability: out of the 23 acceptable questions, 9 were judged as likely to be rhetorical (median ≥ 5), while 8 were rated as likely to be non-rhetorical (median ≤ 3). To quantify the relation between acceptability and rhetoricity, we calculated the correlation using the Pearson correlation coefficient and found a weak correlation between acceptability rating and rhetoricity rating, which was not significant (*r* = 0.23, *p* = 0.066). This finding suggests that rhetoricity is only one of several contributing factors to high acceptability ratings in questions. The results of Experiment 2 indicate that NPIs can appear in rhetorical as well as non-rhetorical questions. Whether there is an underlying factor that determines which NPIs can appear in questions, or whether the acceptability of NPIs in questions is simply determined in the lexicon, needs to be established in future research.

As for the role of minimizer NPIs (marked in red boxes in Fig. 3), this was also smaller than predicted. Within the questions with a high acceptability and rhetoricity rating, only one NPI (*auch nur irgendein* lit.: also only anyone ‘any whatsoever’) is a minimizer NPI. There are two more minimizer NPIs within the questions with a high acceptability rating that received a median rhetoricity rating of 4, which seems inconclusive with regard to rhetoricity: *Menschenseele* (lit.: human.being.soul, ‘living soul’) and *Ton* ‘sound’. There were more minimizer NPIs in the total set of NPIs in Experiment 1 and 2, but since the questions containing these NPIs received acceptability ratings of 4 or lower in Experiment 2, it is difficult to tell their impact on the rhetoricity of the questions. Thus, with regard to the proposal by van Rooy (2003), we did not find exclusively minimizer NPIs in the set of questions with high acceptability and rhetoricity ratings. It seems clear, however, that non-minimizer NPIs can easily occur in rhetorical questions (contra van Rooy, 2003), as we found eight questions with non-minimizer NPIs that were rated as rhetorical in Experiment 2. For example, the question *Kann Caro den Streit mit ansehen?* (*mit ansehen* in Fig. 3, lit. can Caro the fight with behold, ‘Can Caro bear to witness this argument?’) received a median rhetoricity rating of 6. However, the NPI *mit ansehen können* does not denote a minimal amount on any scale. The same goes for the question *Lässt Johannes sich reinreden?* (*reinreden* in Fig. 3, lit.: lets Johannes refl interfere, ‘Is Johannes being swayed on this?’), which received a median rhetoricity rating of 5, and other questions containing verbal NPIs.

Experiment 2 has shown that non-minimizer NPIs can appear in rhetorical questions and minimizer NPIs are not restricted to rhetorical questions. The exact relationship between non-minimizers and the rhetoricity of questions will require further scrutiny in future research: our findings make the applicability of existing theoretical proposals doubtful at best. As for a potential correlation between acceptability and rhetoricity, which we hypothesized to be a reason for the high ratings in the question condition in Experiment 1, the findings of Experiment 2 suggest that this does not exist.

## General Discussion and Conclusion

The two experiments reported in the previous sections aimed at gaining a deeper insight into the licensing conditions of a large set of German NPIs, and at classifying them into superstrong, strong, weak, and superweak NPIs. We tested 60 NPIs and identified seven clusters which group the NPIs according to their acceptability in different licensing contexts. Two of these clusters—[3] and [6]—show acceptability patterns that correspond to the categories which match the predictions from the theoretical perspective of van der Wouden (1994) and Zwarts (1986, 1998). The NPIs in cluster [3] show the licensing pattern of superstrong NPIs, with high acceptability ratings only in the antimorphic condition with the licensor *nicht* ‘not’. The NPIs in cluster [6] can be classified as weak NPIs, with high acceptability ratings in the antimorphic, anti-additive and downward entailing conditions. Some of the NPIs in cluster [6] also received high acceptability ratings in the question condition, hence they can be classified as superweak NPIs. One cluster (cluster [1]) showed low acceptability ratings overall, which likely is due to low frequency/antiquatedness.

There were four clusters of NPIs with less clear licensing patterns. Clusters [2], [5], and [7] exhibit NPI-like behaviour: a drop in acceptability with declining negativity, or in other words, the more negative a context, the higher the acceptability ratings (although in Cluster [2] the non-licensing context did not show very low ratings). The finding that acceptability increases with negativity in principle reflects Zwarts’s (1986, 1998) hierarchy of negation. However, the gradedness is unexpected from the perspective of licensing theories in which an NPI is regarded either as licensed or unlicensed, which should correspond to very high vs. very low acceptability ratings. Graded acceptability effects have also been observed in other experimental work on NPI licensing. For instance, Richter and Radó (2014) found graded acceptability in four experiments, and raise the question of what this entails for theories of licensing. At present, it is not clear how we can account for the gradual differences. The syntactic-semantic approach to NPI licensing does not allow for “half-licensing” by weaker forms of negation. There are theoretical approaches that do not only take syntactic-semantic, but also pragmatic factors into account, for instance Linebarger (1987) and Giannakidou (2006). Pragmatic reasoning in general might be better compatible with graded acceptability. The success of such an account depends on the kind of pragmatic operation that is assumed to be at work. The rescuing approach by Giannakidou (2006) explains why some NPIs can appear in non-downward entailing environments, for example in the scope of non-affirmative (e.g. *to doubt*) or adversative attitude predicates (e.g. *to surprise*): in these environments, NPIs can be rescued by negative inferences that are made available by the aforementioned predicates. Drawing such inferences might be somewhat easier or somewhat harder in different contexts so that gradability is expected. Linebarger (1987) also relies on negative inferences. To what extent such inferences can indeed be applied to the gradual decline of acceptability across gradually reduced negativity that we observed in Experiment 1 needs to be scrutinized in future theoretical work. Questions like how similar the negativity coming with downward-entailing *kaum* ‘hardly’ is to the negativity of anti-additive *kein* ‘no’ in pragmatic terms must be addressed in such a discussion.

Another finding of the current research is that the two nonveridical operators—the question operator and the modal operator *vielleicht* ‘maybe’—show different licensing characteristics. The nonveridical lexical operator *vielleicht* cannot license NPIs in German: throughout, the ratings in the *vielleicht* condition were as low as the ratings in the positive assertion condition. As for the question operator, the combined results of Experiments 1 and Experiment 2 suggest that many NPIs are licensed in questions, and furthermore, that questions are probably not the ‘weakest’ kind of negative context, but are a different kind of licensing context altogether. This tentative conclusion builds inter alia on the licensing pattern of cluster [4] with high acceptability ratings only in the antimorphic and the question condition. Proposals for questions that do not rely on nonveridicality, and thus a superweak degree of negativity, are for instance Kadmon and Landman (1993), Krifka (1994) or van Rooy (2003). Crucially, all these accounts rely on scales. We illustrated this idea for minimizer NPIs in Sect. 4, but it also is applicable for NPIs like *any*. The problem with this is that many NPIs that we found to be acceptable in questions are neither minimizers nor are they scalar elements unless we force very implausible ad hoc scales.

In relation to this, we found that questions are unlikely to be licensing environments due to rhetoricity, as is indicated by the results of Experiment 2. The measure that we used for rhetoricity—how likely participants thought it was that the person asking the question already knew the answer—showed no significant correlation with acceptability. It might be argued that this result should be taken with caution because there is a certain chance that our measure might (also) indicate question bias rather than (or as well as) rhetoricity. Biased questions are similar to rhetorical questions in that they seem to suggest an answer. A biased question like *Isn’t Mark good at statistics?* signals that the speaker has a previous assumption concerning the proposition *p* (that Mark is good at statistics) and double-checks this assumption (= epistemic bias). Biased questions can also be asked when a speaker encounters evidence that challenges their previous assumption (= evidential bias) (e.g., Romero & Han, 2004; Sudo, 2013 et seq.). In Experiment 2, participants could have equated ‘knowing the answer’ with ‘having an assumption about which answer is true’. That is, questions with a high rhetoricity rating were possibly not always interpreted as rhetorical questions, but as questions with an epistemic or evidential bias, where the speaker leans towards one of the possible answers. Item analysis showed that intuitively, some of the questions with a high rhetoricity rating might have been interpreted as a biased rather than a rhetorical or, indeed, a neutral question. The question *Hat Julia auch nur irgendeine Idee?* (lit.: has Julia also only any idea ‘Does Julia have any idea whatsoever?’) from Experiments 1 and 2, for example, seems to signal that the speaker thinks it more *likely* that Julia does not have an idea (= biased question), rather than wanting to state that Julia does not have an idea (= rhetorical question). With the measure employed in Experiment 2, we cannot distinguish biased and rhetorical questions with certainty. However, in view of the role that van Rooy (2003) assigns to NPIs in rhetorical questions, this state of affairs is not really problematic because the difference between the two questions from a pragmatic point is very small: both indicate that the speaker has an answer in mind. How exactly NPIs come into play here needs to be explored in future research—recall that the results of Experiment 2 also indicate that the particular kind of NPI (minimizer or not) neither seems to correlate with acceptability nor with rhetoricity.

Finally, the current research has also shown that individual NPIs seem to have highly idiosyncratic licensing characteristics. An advantage of the rigorous experimental design that we applied, which included the construction of items according to given syntactic patterns, has revealed that some items are sensitive to where exactly their licensor occurs—in the subject position or in the object position. We saw this for the *kein* group. Also recall our discussion concerning the licensor *kaum* ‘hardly’, which may occur as an adverbial quantifier or as a quantificational determiner. Many of these effects might be related to frequency of use, but these issues can and must be explored in future research. Furthermore, detailed experimental testing is needed in order to eliminate some of the noise in the data. Recall that due to the large scale of NPIs tested in Experiment 1, all NPIs were only tested in one lexicalisation. Systematic testing of these NPIs in different lexicalisations is required to arrive at a truly reliable data base, which also includes systematic information on inter-individual variation that could not be tested in the present paradigm because each participant only judged three items per condition (and one per NPI), which was a prerequisite for carrying out a large-scale explorative experiment on the landscape of German NPIs.

In sum, our research has shown that several claims in the theoretical literature can be corroborated with the experimental method that we employed—acceptability judgements for sentences containing NPIs in rich linguistic contexts. We found NPIs that are superstrong, we found NPIs that are weak, and some that are superweak. However, in the cluster analysis, we did not find four clear-cut classes as predicted by the Zwarts (1986, 1995, 1998) and van der Wouden (1994). As already discussed, to a certain degree, that is to be expected when employing experimental methods. However, it is also in line with other empirical work, such as Hoeksema (2012), where 12 different licensing patterns of NPIs were found in a corpus study. It is important to highlight that the present study has not looked at all environments which are known to be NPI licensing, for example at conditionals, but also at presuppositional contexts like superlatives and *only*. Taking these contexts into account in systematic experimental testing of German NPIs is a task for future research. It is then to be expected that the clusters found in Experiment 1 might split up and form different patterns, since more licensing contexts are taken into account. This brings us to the long-standing question of how many classes of NPIs should be assumed: while in the present paper, we have adopted the view of Zwarts (1986, 1995, 1998) and van der Wouden (1994), assuming four classes of NPIs, other researchers only assume two classes of NPIs: weak and strong (e.g., Gajewski, 2011). The data from Experiment 1 as well as Hoeksema’s (2012) corpus data, however, indicate that NPIs cannot simply be classified into two, or even four groups with regard to their licensing behaviour. How many classes of NPIs we should assume once a large number of NPIs as well as all known licensing contexts are factored in on a broad empirical basis is an open question.

Another finding of Experiment 1 was that nonveridicality as such does not seem to be a licensor in German, at least not for the modal operator *vielleicht* ‘maybe’. For other NPIs unpredicted patterns were found which raise important theoretical issues. These concern differences between neutral, biased and rhetorical questions as licensing contexts, and the issue of graded acceptability in relation to graded negativity. The current findings serve as a basis for future research on the licensing of German NPIs in a landscape that is vast and goes beyond ‘classical’ NPIs like *any*, *ever* and *lift a finger*.

## Appendix

**Table:**

Item no	NPI	English translation
Item 01	sonderlich	particularly
Item 02	beileibe	by no means, at all
Item 03	so recht	that well
Item 04	überhaupt	at all
Item 05	unbedingt gern	necessarily, lit. 'necessarily willingly'
Item 06	beim besten Willen	with the best will
Item 07	im Entferntesten	in the slightest
Item 08	im Geringsten	in the slightest
Item 09	brauchen (zu)	need
Item 10	anders können	to be able to act differently, lit. different can
Item 11	Aas	(not) a soul, lit. carrion
Item 12	Deut	brass farthing
Item 13	Geringerer	less a figure, lit. lesser
Item 14	Menschenseele	living soul, lit. human soul
Item 15	Pieps	beep
Item 16	Sterbenswörtchen	dying word
Item 17	Ton	sound
Item 18	Tropfen	drop
Item 19	das Geringste	the least
Item 20	bei Trost sein	to be in one’s right mind, lit.: with consolation be
Item 21	von Pappe sein	to be of sound quality, lit.: of cardboard be
Item 22	von schlechten Eltern sein	to be alright, lit. to be of bad parents
Item 23	auszudenken (sein)	to be conceivable, lit.: make.up be
Item 24	zu übersehen sein	to be conspicuous, lit. to overlook be
Item 25	sich (etwas) befehlen lassen	to be bossed around, lit. refl sth. order let
Item 26	sich (d)reinreden lassen	to be swayed,lit.: refl interfere let
Item 27	sich beirren lassen	to let oneself be misled, lit. refl mislead let
Item 28	sich deuteln lassen	to leave room for interpretation, lit.: to interpret be vs. refl interpret let
Item 29	sich lumpen lassen	to splash out (no literal reading for the verb lumpen available synchronically), lit. refl lumpen let
Item 30	sich rütteln lassen	there is (no) doubt about sth., lit. itself shake let
Item 31	sich unterkriegen lassen	(with negation) to keep one's chin up, lit. refl get.down let
Item 32	es sich nehmen lassen	(with negation) to insist on sth., lit. it refl withdraw let
Item 33	anhaben können	to be able to harm, lit. on.have can
Item 34	absprechen können	not to be able to deprive sb. of sth., lit. deny can
Item 35	ausstehen können	to be able to stand sth./sb., lit. stand can
Item 36	enden wollen	(with negation) to go on and on, lit. end want
Item 37	etwas fassen können	to be able to grasp sth., lit. grasp can
Item 38	etwas wahrhaben wollen	to want to admit sth
Item 39	hinwegtäuschen können	to be able to obscure the fact that …, lit. deceive can
Item 40	lassen können von	can help doing, lit. let can from
Item 41	missen wollen	to want to miss, lit. miss want
Item 42	mit ansehen können	to be able to bear sth., lit. with behold can
Item 43	verwundern können	to be able to amaze sb., lit. amaze can
Item 44	mehr hören können	to be able to hear any longer, lit. any.longer hear can
Item 45	verdenken können	to hold sth. against sb., lit. hold.against can
Item 46	sich erwehren können	to be able to avoid sth., lit. refl to ward off can
Item 47	nachstehen (in)	to be inferior, lit. to after.stand in
Item 48	scheren um	to care about sth./sb., lit. refl care about
Item 49	anfechten (etwas ficht jemanden an)	to irritate, lit. on.fence
Item 50	verhehlen	to disseble
Item 51	nutze / nütze sein zu	to be of any use, lit. useful be to
Item 52	zu besagen haben	to mean sth
Item 53	zu suchen haben	to have no business to be somewhere, lit. to search have
Item 54	zur Sache tun	to matter, lit.: to.the thing do
Item 55	gehen über	to go above
Item 56	dafürkönnen	to be one's fault
Item 57	Abbruch tun	to spoil sth., lit. breaking.off do
Item 58	von ungefähr kommen	to happen by accident, lit. from roughly come
Item 59	(k)einen Millimeter bewegen	to budge an inch, lit. to move (no) millimeter
Item 60	auch nur irgendein	any whatsoever, lit.: also only anyone

## Footnotes

1 Throughout this paper, NPIs in examples will be in bold print, and their licensors will be underlined.

2 It is possible to combine the sentential negation marker *nicht* ‘not’ with the word *ein,* but in these cases *ein* is used as the number word *one*, and the interpretation changes.

3 As mentioned in footnote 2, the NPIs of the *kein* group can only felicitously appear under the scope of *nicht* if the indefinite article is stressed, thus becoming a numeral (*nicht EIN Tropfen* ‘not one drop’). As the experiment was a written questionnaire we did not use test sentences whose acceptability might rely on a ‘special’ intonation pattern, so did not use this possibility to embed the NPIs of the *kein* group under *nicht*.

4 The list of the 60 NPIs tested in the study including contexts, conditions and individual results for both experiments reported are available here: https://osf.io/5vsgj/ (https://doi.org/10.17605/OSF.IO/5VSGJ).

5 We thank an anonymous reviewer for pointing out that the difference between *kaum* as a quantificational determiner and an adverbial quantifier does not necessarily need to be interpreted as involving a semantic distinction between quantification over individuals vs. situations. Yurchenko et al. (2013) found a similar difference for Dutch *bijster* and attributed it to a locality effect.
